# Genetic control of kernel compositional variation in a maize diversity panel

**DOI:** 10.1002/tpg2.20115

**Published:** 2021-07-01

**Authors:** Jonathan S. Renk, Amanda M. Gilbert, Travis J. Hattery, Christine H. O'Connor, Patrick J. Monnahan, Nickolas Anderson, Amanda J. Waters, David P. Eickholt, Sherry A. Flint‐Garcia, Marna D. Yandeau‐Nelson, Candice N. Hirsch

**Affiliations:** ^1^ Department of Agronomy and Plant Genetics University of Minnesota St. Paul MN 55108 USA; ^2^ Department of Genetics, Development, and Cell Biology Iowa State University Ames IA 50011 USA; ^3^ Department of Ecology, Evolution, and Behavior University of Minnesota St. Paul MN 55108 USA; ^4^ PepsiCo R&D St Paul MN 55108 USA; ^5^ United States Department of Agriculture Agricultural Research Service Columbia MO 65211 USA

## Abstract

Maize (*Zea mays* L.) is a multi‐purpose row crop grown worldwide, which, over time, has often been bred for increased yield at the detriment of lower composition grain quality. Some knowledge of the genetic factors that affect quality traits has been discovered through the study of classical maize mutants; however, much of the underlying genetic control of these traits and the interaction between these traits remains unknown. To better understand variation that exists for grain compositional traits in maize, we evaluated 501 diverse temperate maize inbred lines in five unique environments and predicted 16 compositional traits (e.g., carbohydrates, protein, and starch) based on the output of near‐infrared (NIR) spectroscopy. Phenotypic analysis found substantial variation for compositional traits and the majority of variation was explained by genetic and environmental factors. Correlations and trade‐offs among traits in different maize types (e.g., dent, sweetcorn, and popcorn) were explored, and significant differences and meaningful correlations were detected. In total, 22.9–71.0% of the phenotypic variation across these traits could be explained using 2,386,666 single nucleotide polymorphism (SNP) markers generated from whole‐genome resequencing data. A genome‐wide association study (GWAS) was conducted using these same markers and found 72 statistically significant SNPs for 11 compositional traits. This study provides valuable insights in the phenotypic variation and genetic control underlying compositional traits that can be used in breeding programs for improving maize grain quality.

## Core Ideas

- Understanding kernel compositional variation is critical for food‐grade corn applications.
- Genetic and environmental factors account for most of the variation in compositional traits.
- A broad range in trait heritabilities was observed across compositional traits.
- Compositional trade‐offs are important to consider when conducting multiple‐trait breeding.
- Compositional traits are mostly controlled by a large number of small effect loci.

**Abbreviations**
BLUP: best linear unbiased prediction
G×E: genotype × environment
GWAS: genome‐wide association study
MLM: mixed linear model
MLMM: multiple‐locus linear mixed model
NIR: near‐infrared
PVE: phenotypic variance explained
QTL: quantitative trait loci
RNA‐seq: RNA sequencing
SNP: single nucleotide polymorphism
WiDiv: Wisconsin Diversity

## 1 INTRODUCTION

Maize (*Zea mays* L.) is a multi‐purpose crop with a wide variety of uses including feed, fuel, food, and industrial products. In the United States, the vast majority of grain harvested does not contribute directly to human consumption or food products. In 2019, it was estimated that only ∼3.5% of the grain harvested was used for cereals and other food products (USDA–National Agricultural Statistical Service, 2019). In the United States, demand and consumption of maize‐based food products has tripled since the 1970s, primarily through tortillas and tortilla chips as a result of the popularity of both Hispanic and gluten‐free food products (Scott et al., 2019). Maize is also a staple food crop for many other regions around the globe including Latin America and Africa. To meet increasing market demands in the United States and globally, the focus on grain quality in maize breeding programs has become paramount.

Compositional traits are important factors in determining the overall quality of the grain and, in turn, food production. However, in the United States, much of the focus of maize breeding has been on yield gain while neglecting quality traits through much of the breeding pipeline (Holmes et al., 2019). This is quite evident when looking at kernel composition over time in the United States. Investigating commercial hybrids from 1960 to 1990, Duvick and Cassman (1999) found that grain protein content has steadily decreased, and starch content has increased in a trade‐off to select higher yielding hybrids. This change is a negative consequence for the cooking process known as nixtamalization, in which maize kernels are cooked in an alkaline solution to facilitate the removal of the pericarp and are ground into masa dough (Serna‐Saldivar & Rooney, 2015). For example, dry matter loss during cooking is a very important trait to commercial producers as it adds to the production cost of making food products. Pflugfelder et al. (1988) found that hybrids with softer crown and endosperm in the kernel as a result of higher starch content have a higher amount of dry matter loss. The reduction in kernel hardness can also cause a higher level of kernel cracking or stress cracks during storage and drying of the grain at harvest, which leads to increased moisture uptake in the kernel (Jackson et al., 1988) with subsequent impacts on final product quality.

To shift the focus of breeding programs to improved quality traits as a primary breeding objective, a deep understanding of the genetic factors that contribute to variation for compositional traits is beneficial. Much of the current knowledge on the genetics of quality traits comes from the study of classical mutants in maize. Some examples of known mutants include *sugary1* (*su1*) (James et al., 1995), *sugary2* (*su2*) (Zhang et al., 2004), and *waxy1* (*wx1*) (Tsai, 1974), which are involved in the starch biosynthesis pathway and are critical for sweet corn breeding. The *opaque2* (*o2*) (Mertz et al., 1964), *floury2* (*fl2*) (Nelson et al., 1965), and *floury1* (Holding et al., 2007) mutants affect the amino acid content and zein protein composition in the kernel. The *o2* mutant has been used in quality protein maize breeding programs to increase lysine and tryptophan content in animal feed from maize, with much of the work being done by the International Maize and Wheat Improvement Center (CIMMYT) (Babu et al., 2005). The use of these mutations has been difficult to deploy because of the negative effects on yield (Prasanna et al., 2001). Furthermore, long‐term studies, such as the Illinois Long‐Term Selection experiment, on oil and protein content in kernels suggests the genetic control of these traits is quite complex with many small‐effect loci contributing to phenotypic variation (Dudley & Lambert, 2004).

**Core Ideas**

- Understanding kernel compositional variation is critical for food‐grade corn applications.
- Genetic and environmental factors account for most of the variation in compositional traits.
- A broad range in trait heritabilities was observed across compositional traits.
- Compositional trade‐offs are important to consider when conducting multiple‐trait breeding.
- Compositional traits are mostly controlled by a large number of small effect loci.

Genome‐wide association study (GWAS) is a powerful tool that allows researchers to identify regions of the genome that correlate with a trait of interest and determine the genetic control of a trait within a particular germplasm set. The resolution of association mapping depends on the historical recombination that, over time, produces smaller linkage blocks that can be associated with traits (Zhu et al., 2008). A limited number of GWAS have been performed for kernel compositional traits in maize. For example, protein, starch, and oil estimates generated from near‐infrared (NIR) spectroscopy prediction equations in the maize nested association mapping population (McMullen et al., 2009; Yu et al., 2008) and a panel of 302 diverse inbred lines (Flint‐Garcia et al., 2005) found 26, 21, and 22 quantitative trait loci (QTL) respectively through joint‐linkage mapping (Cook et al., 2012). A study of oil and fatty acid composition measured in the AM508 diversity panel of tropical, subtropical, and temperate inbred lines (Yang et al., 2011) revealed 74 QTL underlying these traits (Li et al., 2013). A subsequent study of this same diversity panel detected 247 and 281 statistically significant QTL for 17 proteinogenic amino acid traits in two separate environments (Deng et al., 2017). Finally, the genetic control of carotenoid‐related traits was explored in CIMMYT's 380 inbred carotenoid association panel, and 40 QTL were identified for these traits (Suwarno et al., 2015), and Wang et al. (2020) identified *vitreous endosperm 1* (*ven1*) as a major QTL for influencing vitreous endosperm in the kernel.

Although there have been a handful of studies examining compositional traits in large germplasm panels, there is still a need for better understanding of the compositional trade‐offs between traits as well as their underlying genetic control in order to breed for enhanced composition important for human consumption and food products. Here, we evaluated the Wisconsin Diversity (WiDiv) population (Hansey et al., 2011; Hirsch et al., 2014; Mazaheri et al., 2019), across five environments and evaluated 16 compositional traits obtained from NIR spectroscopy prediction equations to investigate (a) compositional trade‐offs within the population, (b) the role of genotype × environment (G×E) interaction in trait variation, (c) how variation partitions differently across sets of germplasm that are adapted to grow in the upper Midwest, and (d) the genetic control of these traits.

## 2 MATERIALS AND METHODS

### 2.1 Germplasm and genomic data

A subset of 501 lines from the full WiDiv population was used for this study (Supplemental Table S1). These lines all have available whole‐genome resequencing data with ∼20× sequence depth, and SNPs from these data were previously identified (O'Connor et al., 2020). Briefly, reads were aligned to the B73 v4 reference genome assembly (Jiao et al., 2017), and joint SNP calling was performed using freebayes v1.3.1‐17 (Garrison & Marth, 2012).

The SNP calls generated from freebayes were converted to HapMap format with the software package TASSEL version 5.2.64 (Bradbury et al., 2007). Within TASSEL, an overall genetic summary of genotype and site information was obtained. A pairwise genetic distance matrix was calculated by subtracting identity by state from 1 to create a dendrogram that was exported in Newick Tree format, which was visualized using Figtree version 1.4.4 (Cummings, 2014). The resulting dendrogram was visually inspected for consistency with known pedigree relationships and shared heterotic groups. Samples with >15% heterozygosity and SNPs with >15% heterozygosity and 25% missing data were removed using the TASSEL filter menu for sites and taxa. This pruning resulted in the removal of six genotypes (PHJ90, Pa778, N534, 4N506, Va22, and PHR63) and 733,462 SNPs, resulting in a 2,412,791 final SNP set (Supplemental File S1).

The SNP calls from the genomic resequencing data were compared with previous RNA‐sequencing (RNA‐seq)‐generated SNPs on the WiDiv association panel (Hirsch et al., 2014; Mazaheri et al., 2019). Pairwise genetic distance matrices were made for both datasets of common genotypes. A scatterplot comparing the genetic distances of the datasets was used to identify genotypes with inconsistencies between these two data sets (Supplemental Figure S1). Four additional samples (PHR55, B91, PHG86, and B14A) were removed following this criteria. A dendrogram with this final set of genotypes and SNPs was once again generated using the same methods described above (Supplemental Figure S2).

### 2.2 Field experimental design and phenotypic data collection

The 501 genotypes were grown in field trials in the summers of 2016 and 2017. All experiments were planted as a randomized complete‐block design with two replications at each location. Within replicates, genotypes were further blocked by flowering time. The first block consisted of the earlier flowering lines (flowering at 71–80 d after planting based on a previous planting of this population in St. Paul, MN), while the second block consisted of the later‐flowering lines (flowering at 80–87 d after planting). Inbred lines B73 and PH207 were planted as checks within each block, with five entries of each check per block. Plots were grown as single rows (3.35 m long and 0.76 m apart) at ∼70,000 plants ha^−1^. In the summer of 2016, trials were grown at the Minnesota Agricultural Experiment Station (St. Paul, MN) and the Iowa State University Agricultural Engineering and Agronomy Research Farm (Boone County, IA). The trial in St. Paul was planted on 17 May, and ears were harvested 19–25 October. The trial in Boone County was planted on 16 May, and ears were harvested 16–19 October. In 2017, trials were grown again at the Minnesota Agricultural Experiment Station, the Iowa State University Agricultural Engineering and Agronomy Research Farm, and also at the Bradford Research Center (Columbia, MO). The trial in St. Paul was planted on 11 May, and ears were harvested 24–25 October. The trial in Boone County was planted on 8 May, and ears were harvested on 27 September and 4 October. The trial in Columbia was planted on 15 May, and ears were harvested on 28 September and 12 October.

Ears from 10 representative plants in the middle of each plot were harvested and a random sample of 120 grams of kernels was ground using a Mill Feeder 3170 (Perten Instruments). Ground samples were scanned using a Perten DA 7250 NIR spectroscopy scanner, and global prediction equations developed by Perten Instruments (www.perten.com) were used to predict ash as is, fat as is, Fiber as is, moisture, protein as is, and starch as is. ‘As is’ is defined as the trait value without correction for grain moisture at the time of the test. These global prediction equations are developed from a mixture of methods and instrumentation from various laboratories making them more robust than equations developed from a single method (D.E. Honigs, Perten Instruments, personal communication, 2020). Local equations were also developed for the Perten DA 7250 NIR scanner for 10 additional traits with wet chemistry run at Eurofins Scientific, Inc. (Des Moines, IA) using AOAC and AOCS standard protocols: ash (AOAC, 2019a), Ankom crude fiber (AOCS, 2020a), crude fat (AOAC, 2019b), crude fiber (AOAC, 2019c; AOCS, 2020b), fructose (AOAC, 2019d), glucose (AOAC, 2019d), N combustion (AOAC, 2019e, 2019g; AOCS, 2020c), Kjeltec nitrogen (hereafter referred to as N Kjeltec; AOAC, 2019f), sucrose (AOAC, 2019d), and total sugars (AOAC, 2019d). Ankom crude fiber was measured by an Ankom fiber analyzer. Each sample was run in triplicate for equation development. These equations were developed using a set of 100 spectrally diverse inbred lines from the WiDiv association panel (Supplemental Table S2). Wet‐lab data were calibrated to the Perten DA 7250 NIR scanner via the Honigs regression method within the Perten NIR software (Supplemental Figure S3) (Honigs et al., 1985). Raw data for the six global equations and 10 local equations on the 4,886 plots are contained in Supplemental Table S3. It should be noted that for both the global and local equations, some negative values are observed because these values are predictions from regression equations.

### 2.3 Statistical analysis

Statistical analyses were conducted using R version 4.0.2 (R Core Team, 2020). Raw data were plotted for each trait as a whole and by environment to visually identify outliers. Extreme outliers were removed from the dataset if points were beyond five standard deviations from the mean of a given trait, which resulted in the removal of two data points from downstream analyses and from Supplemental Table S3. Each compositional trait was then analyzed using a random‐effects model $y=\mu +g+e+r\left(e\right)+b\left[r(e)\right]+\left(g\times e\right)+\varepsilon$, where *y* is the phenotypic response, *g* is the genotypic effect of an inbred line, *e* is the environmental effect of the year × location combination, *r*(*e*) is the effect of replicate within environment, *b*[*r*(*e*)] is the effect of block nested within replicate nested within environment, *g* × *e* is the genotype × environment interaction, and ε is the residual. Models were fit with the restricted maximum likelihood method using the R package lme4 (Bates et al., 2014), and no violations were detected while checking model assumptions (independence of residuals, residual normality, and homoscedastic variance of residuals) (Supplemental Figure S4).

Heritability was calculated on a line‐mean basis for each trait on an entry‐mean basis using the following equation:

$$h^{2}=\frac{\sigma_{g}^{2}}{\sigma_{g}^{2}+\frac{\sigma_{g\times e}^{2}}{e}+\frac{\sigma_{\varepsilon }^{2}}{re}}$$

where $\sigma_{g}^{2}$ is the genotypic variance, $\sigma_{g\times e}^{2}$ is the genotype × environment interaction variance, $\sigma_{\varepsilon }^{2}$ is the error variance, *e* is the number of environments, and *r* is the number of replications. Pearson correlations between NIR compositional predictions were calculated using the cor function in R. Correlations were conducted on the data set as a whole and then partitioned out based on heterotic groups and types of corn (e.g., dent, sweet corn, and popcorn). Meaningful correlations were interpreted based on the magnitude of the correlation with a threshold of ±0.20, rather than the significance of the correlation as previously suggested (Taylor, 1990; Kozak et al., 2012).

### 2.4 Genome‐wide association analysis

To investigate whether compositional traits should be analyzed separately for each environment or combined across environments, Spearman rank correlations were calculated between environments. Spearman rank correlations revealed that the environments were significantly correlated with each other (*p* < .05) and thus environments were combined. Best linear unbiased predictions (BLUPs) were extracted as the genotypic random effect from the model for each trait. All BLUPs can be found in Supplemental Table S4.

The HapMap data generated in TASSEL was transformed into numerical format in R version 4.0.2 (R Core Team, 2020) using the package GAPIT version 3 (Lipka et al., 2012) by setting the numerical function flag equal to TRUE. GAPIT was also used to generate principal components to be used as covariates in GWAS (Supplemental Figure S5). The GWAS for the WiDiv BLUPs was conducted in R version 4.0.2 (R Core Team, 2020) using the multiple‐locus linear mixed model (MLMM) in the package FarmCPU version 1.02 (Liu et al., 2016). In the model, the first five principal components were included as covariates to account for population structure (Supplemental Figure S5, Supplemental Table S1), and a threshold for initial SNP inclusion into the model was set for each trait by allowing a false entry rate of 1% based on 100 permutations. The SNPs with minor allele frequency <0.05 were discarded, resulting in a final SNP total for the GWAS analysis of 2,386,666. Optimal bin size and quantitative trait nucleotide number were selected for each trait by selecting ‘optimum’ under the method.bin parameter. The SNPs were deemed statistically significant with a Bonferroni‐adjusted *p* value of <.05. A GWAS was conducted using 446 samples (out of the initial 501) after the removal of inbreds with high heterozygosity (6 genotypes), discordance with previous RNA‐seq data (4 genotypes), and lack of genomic sequence data (45 genotypes).

FarmCPU was chosen as the preferred method to conduct GWAS for a few reasons. First, a major issue with GWAS is that many false positives and false negatives appear in the results. To mitigate this issue, kinship and population structure of the association panel are often used as covariates in a mixed linear model (MLM) to control false positives, which, in turn, weakens the signals of the associated SNPs. FarmCPU uses a MLMM that incorporates multiple markers as covariates in a stepwise MLM to limit confounding markers and kinship (Liu et al., 2016). A complete elimination of any confounding from markers and kinship is achieved through the use of a MLMM in two parts in an iterative fashion with a fixed‐effects model and a random‐effects model, which result in increased statistical power, computational efficiency, and control for both false positives and negatives (Liu et al., 2016). Kaler et al. (2020) conducted a study of simulated soybean [*Glycine max* (L.) Merr.] and maize data comparing different statistical models used in GWAS and found that FarmCPU was able to control for false positives and false negatives and was consistently able to identify statistically significant SNPs closest to known published genes. It was also concluded that FarmCPU is a robust model for GWAS of complex traits in plants (Kaler et al., 2020).

Candidate genes for statistically significant SNP hits corresponded to physical positions based on annotation of the AGPv4 reference assembly of inbred B73 (Jiao et al., 2017). Functional annotations of candidate genes were obtained from MaizeGDB (Andorf et al., 2016), BLAST alignments to *Z. mays* (Madden, 2013), and Pfam domains (Finn et al., 2016).

Phenotypic variance explained (PVE) by the 2,386,666 SNPs used for GWAS was calculated as previously described using the software GCTA version 1.93 (Yang et al., 2010). Briefly, in TASSEL version 5.2.64 (Bradbury et al., 2007), SNPs were forced into a biallelic state and then the HapMap format was exported into PLINK ped and map files. In PLINK version 1.07 (Purcell et al., 2007), the two files were used to create the binary ped files used in GCTA. In GCTA, the genome‐based restricted maximum likelihood pipeline was used to calculate the genetic relationship matrix for all SNPs, and PVE was estimated for all 16 traits based on the BLUPs generated from the random‐effects model as the phenotypic input file. In genome‐based restricted maximum likelihood, SNPs are fit jointly in a random‐effect model, and the only estimates of the model are the variance components, which is much smaller than the sample size so there is no overfitting.

### 2.5 Code availability

Code for statistical analysis and GWAS can be found at https://github.com/HirschLabUMN/GWAS_composition.

## 3 RESULTS AND DISCUSSION

### 3.1 Substantial variation for compositional traits exists within the WiDiv association panel

**FIGURE 1:**
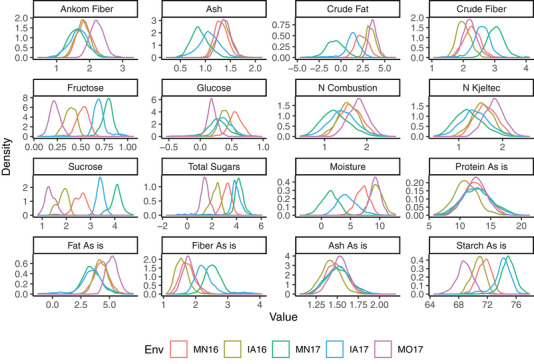
Trait distributions. All 16 near‐infrared prediction trait raw value distributions in each of the five unique environments in 2016 and 2017. The density plot lines are colored according to the corresponding environment. Env, environment; MN16, St. Paul, MN, 2016; IA16, Ames, IA, 2016; MN17, St. Paul, MN, 2017; IA17, Ames, IA, 2017; MO17, Columbia, MO, 2017

The NIR predictions for 16 traits were collected for 501 inbred lines in the WiDiv association panel grown in two environments in 2016 (Minnesota and Iowa) and three environments in 2017 (Minnesota, Iowa, and Missouri). Visualization and descriptive statistics of raw values for compositional traits of the association panel show substantial variation for each trait roughly following normal distributions (Table 1, Figure 1). The variation in compositional traits across this panel of lines is consistent with many other traits that have been previously measured in the WiDiv association panel such as flowering time (Hansey et al., 2011), tassel morphology (Gage et al., 2018), and stalk characteristics (Mazaheri et al., 2019).

**TABLE 1:** Summary of traits and heritabilities

Trait	Mean ± SD	Range^a^	Heritability^b^
	%		
Ankom crude fiber	1.83 ± 0.32	0.43–3.32	0.92
Ash as is	1.49 ± 0.13	1.06–2.19	0.94
Ash	1.21 ± 0.25	0.22–2.13	0.89
Crude fat	1.99 ± 1.80	(−)4.68–6.24	0.79
Crude fiber	2.42 ± 0.44	1.06–4.1	0.84
Fat as is	4.17 ± 1.00	(–)1.28–7.08	0.88
Fiber as is	1.99 ± 0.45	1.09–4.06	0.84
Fructose	0.52 ± 0.21	0.04–1.06	0.50
Glucose	0.36 ± 0.18	(−)0.54–0.98	0.54
Moisture	6.31 ± 3.31	(−)3.64–12.71	0.84
Nitrogen combustion	1.53 ± 0.33	0.42–2.68	0.94
Nitrogen Kjeltec	1.52 ± 0.33	0.43–2.61	0.94
Protein as is	12.32 ± 2.17	5.75–21.27	0.94
Starch as is	72.01 ± 2.43	64.52–77.82	0.75
Sucrose	2.65 ± 1.01	0.82–4.76	0.44
Total sugars	3.00 ± 1.13	(−)1.69–6.05	0.51

^a^ Negative values are observed as a result of the fact that these values are predictions from regression equations.

^b^ Heritability is calculated on a line‐mean basis.

Means, standard deviations, and ranges were calculated from the raw data across all environments (Table 1). Crude fat had the largest coefficient of variation (CV = 0.90) with values ranging from −4.68 to 6.24%. The smallest CV observed was for starch as is (0.03) with a range of 64.52–77.82%. For the global equation traits, protein as is ranged from 5.75 to 21.27%, fiber as is ranged from 1.09 to 4.06%, fat as is ranged from −1.28 to 7.08%, starch as is ranged from 64.52 to 77.82%, and ash as is ranged from 1.06 to 2.19% (Table 1). The ranges for the global equation traits in the WiDiv association panel are consistent with a previous study that analyzed a 282 inbred panel, in which starch, protein, and oil ranged from 59.6 to 70.3%, 11.5 to 17.5%, and 3.1 to 8.2%, respectively (Cook et al., 2012). Compared with a study by Reynolds et al. (2005), who analyzed seven contemporary maize hybrids, the proximate starch content was higher than in WiDiv (79.3–88.0 vs. 64.52–77.82% in WiDiv) and also had a smaller range in protein (8.03–15.6 vs. 5.75–21.27% in WiDiv). This observation is consistent with a general trade‐off between starch and protein content that has been observed in maize breeding history (Duvick & Cassman, 1999).

The 501 inbred lines of the WiDiv association panel consist of many different types of maize, including 145 stiff stalk, 143 nonstiff stalk, 80 unknown, 54 mixed, 50 Iodent, 12 tropical, 11 popcorn, five sweet corn, and one flint based on pedigree information (Supplemental Table S1). Given the range of types of maize that comprised the subset of the WiDiv panel included in this study, we investigated the differences in composition between types and heterotic groups. The BLUP‐adjusted means were generated from a random effects model for each type and a Tukey's HSD (α = 0.05) was used to investigate if statistically significant differences existed for compositional traits among maize types (Table 2). For starch as is, sweet corn had the lowest value at 70.86% and differed statistically from all other types. This can be attributed to the physiological differences among sweet corn kernels vs. dent kernels, in which sweet corn kernels have been bred to have increased sugar content and less starch content at maturity (Tracy, 2010). Popcorn had the highest amount of Ankom crude fiber, crude fiber, and fiber as is and differed statistically from the dent germplasm, that is, stiff stalk, nonstiff stalk, and Iodent (Table 2). The majority of the fiber in corn kernels is found in the pericarp, which is the outermost layer that encases the entire kernel except the tip cap and is comprised largely of hemicelluloses including xylans and arabinoxylans (Chateigner‐Boutin et al., 2016; García‐Lara et al., 2019). In a study comparing anatomical percentages of kernel anatomy between dent, flint, and popcorn, popcorn had the highest proportion of pericarp at 7% followed by flint at 6.5% then dent at 6.0% (Serna‐Saldivar, 1996). Stiff stalk and Iodent types differed statistically for crude fat from tropical and for fat as is from tropical and sweet corn types, with the lowest values compared with the other maize types. In the kernel, the majority (∼85%) of fatty acids are found in the embryo (Shen & Roesler, 2017). This finding suggests that stiff stalk and Iodent types may have been selected and bred over time to have a smaller proportion of germ (i.e., embryo) and less protein in the endosperm, though not significantly different, which would increase starch content at the cost of protein within the endosperm compared with other maize types. No statistically significant differences between BLUP adjusted means for ash, nitrogen combustion, nitrogen Kjeltec, and protein as is were observed across maize types (Table 2).

**TABLE 2:** Summary of best linear unbiased prediction (BLUP)‐adjusted mean differences for each compositional trait compared among maize types

Trait	Stiff stalk	Non‐stiff stalk	Unknown	Iodent	Mixed	Popcorn	Tropical	Sweet corn	Flint
Ankom crude fiber	1.76c^a^	1.85c	1.90c	1.83c	1.85c	2.19a	1.97bc	2.15ab	2.10abc
Ash	1.17ab	1.23a	1.24a	1.18ab	1.24a	1.28a	1.27a	1.32a	1.43a
Ash as is	1.46b	1.51a	1.54a	1.46ab	1.51a	1.62a	1.58abc	1.69a	1.74a
Crude fat	1.92b	2.06ab	2.15ab	1.86b	2.05ab	2.17ab	2.41a	1.99ab	2.08ab
Crude fiber	2.39c	2.43bc	2.43bc	2.46b	2.42bc	2.68a	2.44bc	2.58ab	2.41bc
Fat as is	4.06b	4.16ab	4.35ab	3.81b	4.20ab	4.50ab	4.65a	4.77a	4.58ab
Fiber as is	1.95d	1.98cd	2.02bc	2.04bc	1.98cd	2.28a	2.02bcd	2.17ab	1.92d
Fructose	0.53a	0.52abc	0.52bc	0.53a	0.52ab	0.50d	0.52bc	0.51cd	0.51bcd
Glucose	0.35bc	0.36b	0.35c	0.38a	0.36bc	0.33c	0.35bc	0.35c	0.35bc
Moisture	6.45ab	6.23bc	6.08cd	6.57a	6.18bcd	5.57d	5.88cd	5.75cd	5.45d
N combustion	1.47ab	1.58a	1.63a	1.43ab	1.59a	1.70a	1.71a	1.78a	2.05a
N Kjeltec	1.45ab	1.56a	1.61a	1.42ab	1.57a	1.69a	1.69a	1.75a	1.99a
Protein as is	11.80ab	12.63a	13.04a	11.61ab	12.74a	13.51a	13.64a	14.47a	15.67a
Starch as is	72.08a	72.03a	71.85a	72.11a	72.02a	71.79a	71.83a	70.86b	72.37a
Sucrose	2.64b	2.64b	2.63b	2.67a	2.64b	2.64b	2.63b	2.65ab	2.64b
Total sugars	2.98bc	2.99b	2.94c	3.09a	2.99bc	2.92c	2.97bc	2.89c	2.95bc

^a^ Type‐adjusted means followed by the same letter within a trait are not statistically different according to Tukey's HSD (p < 0.05).

### 3.2 Genetic and environmental factors account for most of the variation in compositional traits

**FIGURE 2:**
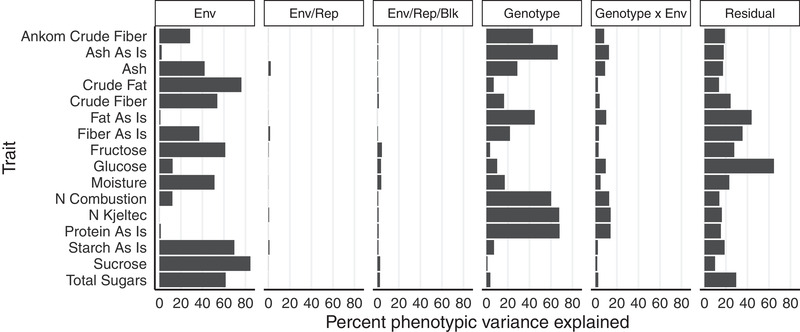
Percentage of phenotypic variance explained by each factor from the ANOVA of the random effects model for 16 compositional traits collected from the Wisconsin Diversity association panel. Env, environment; Rep, replication; Blk, block

Given the high degree of variation observed for all of the compositional traits, we next sought to determine the sources of the variation. The proportion of variance explained by each term from the random effects model was analyzed, and the environment term, which combines year and location, was statistically significant (*p* value < .001) for all traits and accounted for 0.02–84.65% of the total observed variation, with a median of 39.59% (Figure 2; Supplemental Table S5). The environment term explained the majority of the phenotypic variance for ash, crude fat, crude fiber, fiber as is, fructose, moisture, starch as is, sucrose, and total sugars. To investigate the role of a location or year effect on compositional traits, the environment term was partitioned into location and year effects within the random effects model. The location effect ranged from 0 to 72.93%, the year effect ranged from 0 to 4281%, and the location‐within‐year effect ranged from 0 to 77.04% (Supplemental Figure S6). The location effect was the predominant source of variation for Ankom crude fiber, crude fat, fat as is, fructose, and total sugars (Supplemental Figure S6). The year effect explained the most phenotypic variance for glucose. The location‐within‐year effect was the largest for crude fiber, fiber as is, starch as is, and sucrose. For many of the carbohydrate compounds analyzed, the location was the driving factor of the environmental effect, which can also be seen in the distribution of the raw phenotypic data (Supplemental Figure S6). Of the five unique environments, the MO 2017 environment, which is the most geographically and climatically distinct environment, was the lowest for all carbohydrate traits. Previous studies have also found a statistically significant effect of the environment on compositional traits. For instance, oil concentration in the grain has been shown to be greatly influenced by geographic location and planting year (Dunlap et al., 1995; Jellum & Marion, 1966). Likewise, a study of seven maize hybrids grown in Europe analyzing compositional traits found 1,986 statistically significant differences out of 4,935 comparisons between the four distinct environments included in the study, highlighting the importance of environment effects for these traits (Reynolds et al., 2005). These results highlight that environmental factors, such as location and year effects, need to be taken into consideration for breeders to meet breeding targets for compositional traits.

The genotype and G×E interaction terms were also statistically significant for all traits (*p* < .001) and ranged from 1.07 to 68.06% (median 19.49%) and 1.77 to 14.19% (median 6.48%), respectively (Figure 2; Supplemental Table S5). In contrast, a previous study analyzing a different maize association panel found kernel moisture and starch content had a nonsignificant G×E interaction, while oil and protein content did have a statistically significant G×E interaction (Flint‐Garcia et al., 2005). However, in that study the authors noted that many environments had only a single replication, which caused the G×E interaction to be confounded with the residual term in the mixed model, which would affect significance testing. While the G×E interaction was not the main source of phenotypic variation, it was still a statistically significant source of variation for all traits. Breeders have options for dealing with G×E interactions: choose to ignore these interactions and instead choose varieties that are superior when averaged across environments, try to reduce these effects by testing varieties in similar environments, or try to exploit these interactions by selecting varieties that excel in a particular environment (Bernardo, 2010). For Ankom crude fiber, ash as is, fat as is, nitrogen combustion, nitrogen Kjeltec, and protein as is, the genotype term in the model explained the most phenotypic variation, with 43.35–68.06% of the variation explained. While for ash, crude fiber, fiber as is, and moisture intermediate values of variation explained by genotype were observed ranging from 16.43 to 28.82% or low values for the carbohydrate traits ranging from 1.07 to 10.00% (Figure 2). The approach for dealing with G×E interactions will likely need to be different for these different classes of traits.

Finally, the residual error ranged from 9.90 to 64.69% of variance explained and accounted for the highest variance explained for glucose, which had only 10.00% of the variation explained by genotype (Figure 2). The high residual variance for some traits may reflect the lower quality of the local prediction equations for those traits (Supplemental Figure 3) and also the detection limits of analytical techniques specifically in the case of total sugars, glucose, fructose, and sucrose (<0.15%; Supplemental Table S2). Still, a sizable proportion of variance explained was due to residual variation for the rest of the compositional traits. Some of these factors could be epistatic effects or micro‐environmental factors that were not taken into consideration in our random effects model.

### 3.3 Heritabilities of compositional traits and implications for single‐trait breeding targets

Trait heritability is an important consideration in determining the ability to make gains from selection for a particular trait by a breeder. Traits that have a high heritability have better response to selection (Falconer & Mackay, 1996), and understanding the heritability for compositional traits in the breeding population helps breeders plan where to invest or divest resources to make genetic gains for the desired breeding target. Heritabilities on a line‐means for the diversity panel ranged from 0.44 (sucrose) to 0.94 (ash as is, protein as is, nitrogen combustion, and nitrogen Kjeltec) (Table 1). With the exceptions of fructose, glucose, sucrose, and total sugars, the compositional traits measured in this study all had relatively high heritabilities (>0.75) (Table 1), indicating a larger genotypic variance than G×E and residual variances that were measured in multiple environments. A previous study evaluating heritability in a panel of diverse inbred lines (Flint‐Garcia et al., 2005) also found high heritabilities for protein, oil, and starch from NIR predictions that ranged from 0.83 to 0.91 (Cook et al., 2012). The lower heritabilities seen in the sugar‐related traits in our study are likely a reflection of a lower quality prediction model (*r* = 0.260–0.649; Supplemental Figure S3) than the global predictions equations but could also, to some extent, reflect true biological differences in the degree of environmental control of these traits.

The observed variation for compositional traits within the WiDiv association panel (Figure 1) and the relatively high heritabilities (Table 1) are critical for breeders to make gains from selection for quality traits within their breeding programs. The high heritabilities for these NIR predictions indicate that NIR is a suitable, high‐throughput, and affordable method, compared with wet lab techniques, to quickly evaluate germplasm within a breeding program and obtain robust data for making breeding decisions. Ten of the 16 traits showed that the environment was the predominant source of phenotypic variation in the random‐effects model. For these traits, potential improvement could be made by breeding for target environments or growing and sourcing from certain environments that are more favorable for specific traits of interest. Ankom crude fiber, ash as is, nitrogen combustion, nitrogen Kjeltec, and protein as is had the majority of the phenotypic variation partitioned to the genotype term in the random effects model and had very high heritabilities with an average of 0.94 (Table 1). For these traits, breeders can make genetic gains through selection that can be deployed across more diverse environments.

### 3.4 Correlations between compositional traits among types of maize and implications for multiple‐trait breeding

Phenotypic correlations were detected among the WiDiv association panel as a whole (Supplemental Table S6). The strongest correlations were between the various protein and nitrogen measurements, including nitrogen Kjeltec, nitrogen combustion, and protein as is (*r* = 0.99–1.00). Nitrogen combustion is collected via the Dumas method (Dumas, 1831), which determines total nitrogen content (organic and inorganic nitrogen) by combusting a sample at a high temperature in an oxygen‐rich environment to convert the nitrogen in the sample into nitrogen gas. Nitrogen Kjeltec is measured with the Kjeldahl method (Kjeldahl, 1883), which determines the total organic nitrogen content of a sample through a sulfuric acid digestion. Protein as is is then calculated by a protein conversion factor of 6.25 for maize to estimate the total protein content in a sample. The fact that these traits are highly correlated is not particularly surprising, as they are measuring the same compound in different ways, but gives confidence to the quality of the data.

By investigating phenotypic correlations of distinct compounds, we found that protein as is was negatively correlated with starch as is (*r* = −0.23) (Supplemental Table S6). The negative trade‐off between protein and starch throughout the history of maize breeding is well known. Breeding for increased yield in maize has driven an increase in starch content over time with an overall decrease in protein content (Duvick & Cassman, 1999). This is because a lower energy threshold is required of the plant to make starch since it is a primary product of photosynthesis, while protein requires more energy and relies on external factors such as nitrogen availability in the soil (Jenner et al., 1991). In a previous study by Cook et al. (2012) a negative correlation was found between NIR‐predicted starch and protein (*r* = −0.56) in a panel of 282 diverse inbred lines. We also found a negative phenotypic correlation between starch and fat (*r* = −0.46) and a positive correlation between protein and fat (*r* = 0.54), both of which were consistent with Cook et al. (2012). In the maize kernel, fat content is primarily found in the germ, and starch and protein are found in the endosperm. The endosperm is roughly divided into the floury endosperm, which contains mostly starch and is located in the interior of the kernel, and the vitreous endosperm surrounding it, which contains much of the protein found in the kernel (Holmes et al., 2019). For the purpose of food‐grade maize, a shift in the proportion of floury endosperm to vitreous endosperm, or a larger germ relative to the endosperm, will be needed to increase the relative amount of protein and fat in the kernel.

**FIGURE 3:**
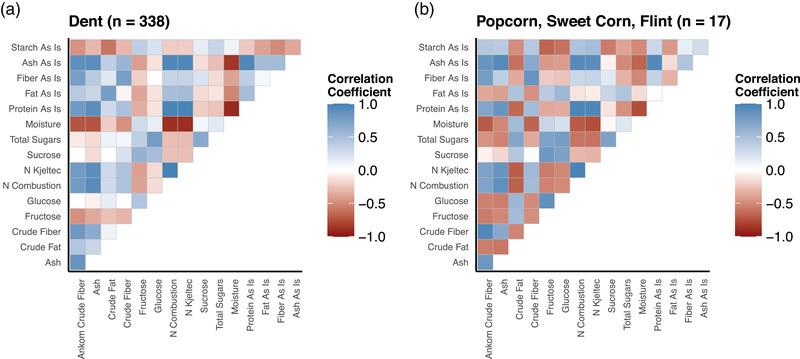
Heatmaps of Pearson correlation matrices for compositional traits partitioned by (a) dent, and (b) popcorn, sweet corn, and flint

Breeding efforts for different applications (e.g., food‐grade corn, sweet corn, popcorn, etc.) are focused on different needs based on the product market, which have resulted in very different trait means (Table 2) and potentially different correlations among traits within these groups of germplasm. Pearson correlations were recalculated after separating the lines into dent (stiff stalk, nonstiff stalk, and Iodent) vs. popcorn, sweet corn, and flint since these two groups showed the most statistically significant differences in BLUP‐adjusted means between traits (Table 2). Overall, more meaningful pairwise correlations were detected for popcorn, sweet corn, and flint than in the dent germplasm (Figure 3). The overall pattern of pairwise correlations were relatively similar, but the changes in magnitude and direction were substantial in some instances (Figure 3). Starch as is had weak positive correlations with glucose, fructose, and sucrose (*r* = 0.23, 0.37, and 0.14, respectively) in pooled dent germplasm, but in popcorn, sweet corn, and flint lines these correlations changed direction and were stronger negative correlations (*r* = −0.61, −0.66, and −0.56, respectively) (Figure 3). Similar changes in correlation patterns were also observed after separating lines into dent and nondent for correlations between crude fat and numerous other traits (Ankom crude fiber, ash, fructose, nitrogen Kjeltec, and nitrogen combustion) as well as the correlation of crude fiber with both moisture and protein. The correlation between protein and crude fat was positive (*r* = 0.28) in the dent germplasm but was reversed to a negative relationship (*r* = −0.65) in the nondent germplasm.

Breeding for a single compositional trait can be a difficult task, given controlling genetic and environmental factors, but when improving multiple traits simultaneously in a breeding program, the task becomes even more complex and difficult. Phenotypic correlations in the full WiDiv association panel and parsing the panel into dent and nondent types revealed that there are many phenotypic trade‐offs that need to be assessed by a breeder in order to make desired genetic gains. For instance, if a breeder has a program goal of improving quality, certain phenotypic correlations may work for or against the breeding target. Protein in the dent germplasm pool was negatively correlated with starch (*r* = −0.23), but positively correlated with ash, fiber, and fat (*r* = 0.96, 0.52, and 0.53, respectively) (Figure 3). Selections for increasing protein content could then also translate to gains in ash, fiber, and fat but a decrease in starch content that would meet the breeding targets of food‐grade maize. These compositional targets help with physiological aspects of the kernel, such as kernel damage, stress cracks, and higher protein content, that impact processing. There are a few methods for multiple‐trait selection such as tandem selection, independent culling, and index selection (Bernardo, 2010). In tandem selection, a breeder selects for a single trait at a time until breeding targets have been met. This method can be time consuming but can be efficient if traits are correlated with each other in a way that meets the breeding targets. Independent culling, on the other hand, selects for multiple traits in a single breeding cycle with minimum threshold levels determined for each trait, and entries that are above these thresholds continue through the breeding pipeline. Index selection selects for multiple traits simultaneously using an index value calculated by the breeder. The index can weight traits based on economic value alone, genetic and phenotypic covariances with economic values, and performance (Elston, 1963; Hazel, 1943; Smith, 1936; Williams, 1962). Index selection is a more effective method than both tandem selection and independent culling while having the ability to have multiple traits imputed into an index. Index selection can also be tailored to a breeding program with multiple methods from which a breeder can choose. For instance, a food‐grade maize breeder with the goal of increased quality would put higher weights on protein, fiber, and fat content with a smaller weight on starch content. Knowledge of how compositional traits interact with each other at a phenotypic level is important for making genetic gains in a breeding program, and understanding the genetic control of these traits can help accelerate breeding goals for improved quality in maize grain.

### 3.5 Compositional traits are mostly controlled by a large number of small‐effect loci

**FIGURE 4:**
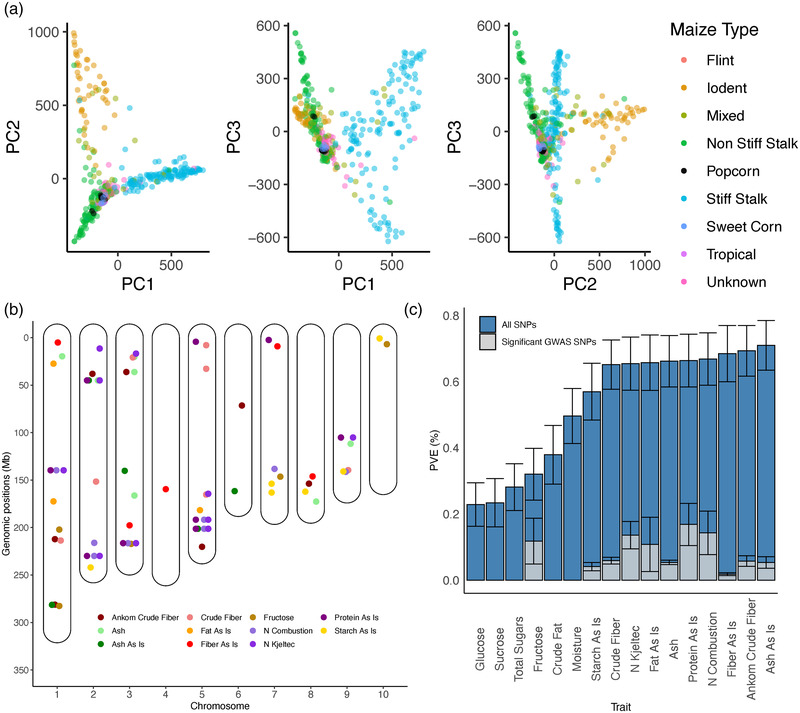
Genetic control of compositional traits in a maize diversity panel. (a) Pairwise plots of the principal components (PCs) of population structure using 2,386,666 single nucleotide polymorphisms (SNPs) for the 446 inbred lines used for genome‐wide association studies (GWAS). Each point is colored based on maize type classification. (b) Statistically significant GWAS SNP associations of compositional traits. Each point represents the physical location of a statistically significant SNP on the chromosome. Points are colored corresponding to a particular compositional trait. (c) Percentage variation explained (PVE) by the full set of 2,386,666 SNPs used in the GWAS are colored in blue, while the gray corresponds to significant SNPs that were specific for each trait based on GWAS results. Error bars represent the standard error

To determine the genetic control of our compositional traits we used a GWAS approach, as this population was originally developed for this purpose (Hansey et al., 2011). For this analysis, we used a set of 2,386,666 SNPs that had been previously called from whole‐genome resequencing data (O'Connor et al., 2020). Principal component analysis using these SNPs showed clear separation of the three major maize dent heterotic groups: stiff stalk, Iodent, and nonstiff stalk (Figure 4a). The patterns observed in the principal component analyses are similar to other published results (Romay et al., 2013; van Heerwaarden et al., 2012; White et al., 2020) describing the separation of maize types. The clear separation of maize types from the whole‐genome resequencing data gave us confidence in the genotypic data and provided covariates to use in the GWAS to account for population structure.

For all 16 compositional traits in this study, the genetic control was mapped using 446 inbreds with resequencing data using the MLMM in FarmCPU (Liu et al., 2016) with 2,386,666 SNPs. A total of 72 statistically significant SNPs across 11 of the 16 traits were detected based on a genome‐wide corrected Bonferroni threshold of −log10(*p*) = 7.68 (Figure 4b; Supplemental Figure S7, Supplemental Table S7). The traits with statistically significant GWAS SNP associations included Ankom crude fiber (7), ash (7), ash as is (5), crude fiber (7), fat as is (3), fiber as is (5), fructose (5), nitrogen combustion (8), nitrogen Kjeltec (10), protein as is (9), and starch as is (6) (Figure 4b). In a previous study conducted by Cook et al. (2012) using NIR‐predicted data for starch, protein, and oil, no significant associations were found using a MLM method for GWAS, which is contrary to our study in which starch, protein, and fat from the global equations had statistically significant SNPs. The differences between the two studies could be due to a few factors, such as having more individuals in the WiDiv association panel, a more balanced allele frequency profile because the WiDiv is less genetically diverse than the 282 panel, a much larger SNP dataset (43 times as many markers), and a more powerful GWAS model to detect significance for these complex compositional traits.

As we had multiple traits in this study that measured the macromolecules in different ways for protein, starch, and ash, we decided to first look at the overlap in hits within these sets of traits within 50 kb. There were eight instances of overlap between compositional traits (Figure 4b; Supplemental Table S7), including SNPs on chromosomes 1, 2, 4, and 5 between nitrogen combustion, nitrogen Kjeltec, and protein as is; SNPs on chromosome 2 between ash, ash as is, nitrogen combustion, and nitrogen Kjeltec; SNPs on chromosome 3 for Ankom crude fiber and ash; SNPs on chromosome 5 between ash as is, nitrogen combustion, nitrogen Kjeltec, and protein as is; and SNPs on chromosome 9 between nitrogen Kjletec and protein as is (Figure 4b). While these overlaps are observed, this does not necessarily indicate that the same causative variant is underlying variation across each of these traits, and it is possible that this observation is the product of variants that are in close physical proximity to each other. However, for the shared statistically significant SNPs between nitrogen combustion, nitrogen Kjeltec, and protein as is, the same exact SNPs was identified. This is not unexpected as these traits are highly correlated (*r* = 1.00, 0.99, 0.99, respectively) with each other and are measuring nitrogen content in slightly different fashions as described above (Supplemental Table S6). While the nitrogen‐related compositional traits shared many SNP hits, the fiber and carbohydrate‐related traits all gave different statistically significant SNPs. This is not particularly surprising, as fiber can be measured in multiple ways reflecting different compounds within the fiber fraction (Mertens, 1992). This is exacerbated when comparing the local equations with the Perten global equations. The local equations were developed with a single method, while the global equations were developed using a mixture of methods that integrated data from different laboratories (D.E. Honigs, Perten Instruments, personal communication, 2020), which may reduce signal gained from using equations developed from a single method and measuring a specific aspect of fiber.

Across the 72 statistically significant SNPs, 48 were located within a gene in the B73v4 annotation, and the remaining 24 were within 18 kb of a gene model (Supplemental Table S7). Functional annotations for the genes nearest each statistically significant SNP were determined by using a series of databases (see Materials and Methods section). Only 13 statistically significant SNPs yielded uncharacterized protein annotations from the combined gene annotation methods (Supplemental Table S7). A notable association for Ankom crude fiber includes a WUSCHEL‐related homeobox gene on chromosome 8, which has previously been shown to be associated with maize embryo development (Nardmann et al., 2007). For fat as is, a bzip transcription factor 111 previously found to be involved in the carbon–nitrogen balance regulation (Wu et al., 2019) was identified as a candidate gene. While these results provide an exciting resource to breeders to make quality gains in maize, and this study identified more statistically significant SNPs than previous GWAS studies of compositional traits in maize (Cook et al., 2012), the SNPs identified in this study do not explain the full breadth of phenotypic variation in this population.

To gain more insight into the underlying genetic control of these compositional traits, we estimated the PVE by the full set of 2,386,666 SNPs generated from the whole‐genome resequencing. The percentage of PVE ranged from 22.88 to 70.98%, with glucose having the smallest amount and ash as is having the most (Figure 4c). The PVE was also calculated for just the significant SNPs identified for each trait using only those SNPs that were significant in the GWAS for the trait. The PVE for these significant GWAS SNPs was substantially lower and ranged from 1.84 to 16.86%, with fiber as is having the smallest PVE from the significant SNPs and protein as is having the most (Figure 4c). The carbohydrate traits tended to have the lowest amount of variation explained, ranging from 22.88 to 57.00% (Figure 4c) vs. the other traits. This could be due to the accuracy of the NIR prediction equations used to generate values for the sugars or reflects the large role that the environment contributes to these traits (Figure 2). Nine of the 16 traits had relatively large (>60%) PVE attributed to the full set of SNPs, indicating that many loci of small effect contribute to variation of compositional traits and did not meet the threshold of significance rather than a few large effect SNPs identified in the GWAS. A previous GWAS study for kernel compositional traits found no significant SNPs in their association panel Cook et al. (2012) but postulated if the population size of the diversity panel increased as well as the SNPs density it would be easier to detect small‐effect loci. This result is consistent with results found in the Illinois long‐term selection experiment in a QTL study for oil concentration, where it was estimated that more than 50 loci may be involved in controlling the trait (Laurie et al., 2004). The upper limits for protein and oil have not been reached in the Illinois long‐term selection experiment even after over 100 generations of selection (Lucas et al., 2013), giving reason to believe many of these small‐effect loci can be used to make gains for improved quality grain for these and other compositional traits. This result brings more complexity to the challenge for breeding to improve for quality traits and the cost of marker data across many small‐effect loci. Multiple strategies are available to breeders today to try and make genetic gain for compositional traits including the production of doubled‐haploid populations, marker‐assisted selection, genomic prediction, and recurrent selection programs in order to harness these small‐effect loci.

## 4 CONCLUSIONS

In summary, we collected NIR compositional traits on a set of 501 inbred lines adapted to the upper Midwest grown in five unique environments. The WiDiv association panel showed substantial variation for compositional traits, and it was shown that genetic and environmental factors account for most of the phenotypic variation observed. Statistically significant differences in means among types of maize and meaningful correlations between compositional traits were found to reflect the diversity of maize types and the underlying anatomical and structural makeup of the kernels. A total of 2,386,666 SNPs derived from whole‐genome resequencing data was used to conduct a GWAS on 446 individuals from the WiDiv association panel, which resulted in 72 statistically significant SNPs detected. The proportion of variance explained by all of the SNPs showed that many compositional traits are controlled by small‐effect loci. Insights into the underlying genetic control of these traits and how they are correlated will help breeders in implementing breeding strategies for quality enhancement breeding targets that have been largely neglected to date.

## CONFLICT OF INTEREST

NA, AJW, and DE are employed by PepsiCo, Inc., a goods and beverage company that sources food grade corn. The views expressed in this manuscript are those of the authors and do not necessarily reflect the position or policy of PepsiCo, Inc.

## AUTHOR CONTRIBUTIONS

CNH, MYN, SFG, DE, AW, NA conceived this experiment. JSR, AMG, TJH conducted the experiments, JSR, CHO, PJM analyzed the data, JSR visualized the data, JSR, CNH wrote the original draft, all co‐authors edited and approved the final manuscript.

## Supporting information


**Supplemental Figure S1**. Genetic distance comparison between shared inbreds for RNA‐seq (Mazaheri et al., 2019) and WGS (O'Connor et al., 2020) data.
**Supplemental Figure S2**. Dendrogram of the 446 inbred lines of the WiDiv association panel with 2,412,791 SNPs from WGS data.
**Supplemental Figure S3**. Comparison of observed and predicted values for local near‐infrared (NIR) spectroscopy prediction equations derived from a subset of 100 inbred lines with diverse spectra profiles.
**Supplemental Figure S4**. Quantile‐quantile (Q‐Q) plots of the sixteen compositional traits for the random effects model. The red line represents the 1:1 y = x line.
**Supplemental Figure S5**. Scree plot of eigen values for the principal components from the whole genome sequencing data used on the WiDiv association panel.
**Supplemental Figure S6**. Percent of phenotypic variance explained by each factor from the ANOVA of the random effects model for sixteen compositional traits collected from the WiDiv association panel.
**Supplemental Figure S7**. Manhattan plots from the GWAS of each compositional trait. Each point represents a single SNP and the solid red line represents the Bonferroni corrected p‐value threshold to deem a statistically significant SNP at the 0.05 level (‐log(p) = 7.68).

**Supplemental Table S1**. Description of the inbred lines within the Wisconsin Diversity (WiDiv) association panel that were included in this study.
**Supplemental Table S2**. Compositional wet lab trait observations used to develop local equations based on 100 spectrally diverse inbred lines from the WiDiv association panel and the predicted trait values from those equations.
**Supplemental Table S3**. Phenotypic data for inbreds of the WiDiv association panel collected at five unique environments.
**Supplemental Table S4**. Best linear unbiased predictions (BLUPs) for compositional traits of inbreds in the WiDiv association panel.
**Supplemental Table S5**. Analysis of variance (ANOVA) from a linear model for 16 compositional traits collected from the WiDiv association panel.
**Supplemental Table S6**. Pearson correlation coefficients between all traits (BLUPs) evaluated in the full WiDiv association panel.
**Supplemental Table S7**. Statistically significant SNP locations and corresponding candidate genes identified through GWAS of 446 inbred lines from the WiDiv association panel for compositional traits.
