# Supplementary material for: Genetic control of kernel compositional variation in a maize diversity panel
Source: Plant Genome. 2021 Jul 1;14(3):e20115. doi: 10.1002/tpg2.20115 (PMC12807035; doi:10.1002/tpg2.20115)
Supplement: Supplementary file 1 [file TPG2-14-e20115-s002.pdf]

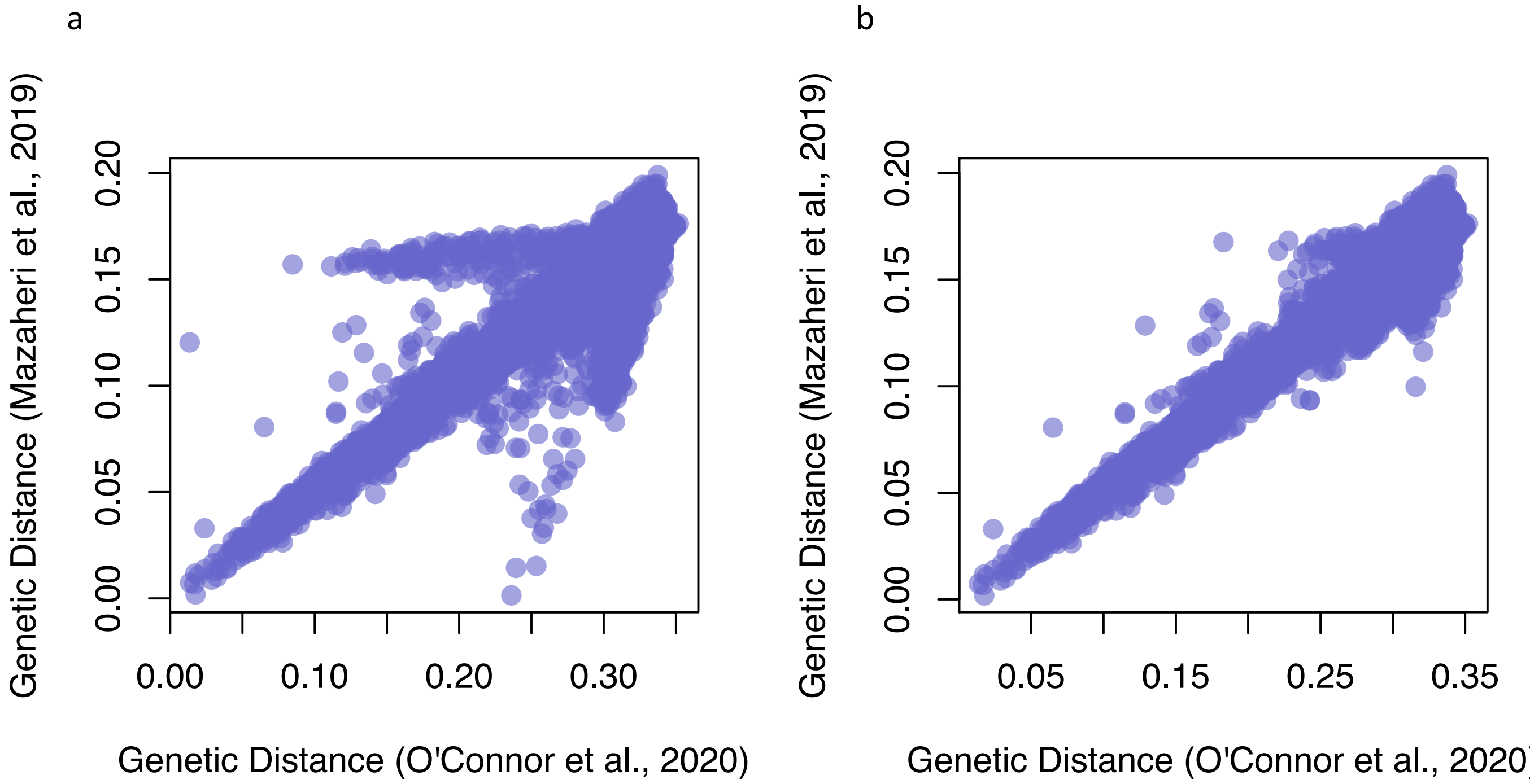

Supplemental Figure 1. Genetic distance comparison between shared inbreds for RNA-seq (Mazaheri et al., 2019) and WGS (O'Connor et al., 2020) data. a) All inbreds included in GD comparison. b) Inbred lines B91, B14A, PHG86, and PHR55 removed due to inconsistencies between genotypic datasets.

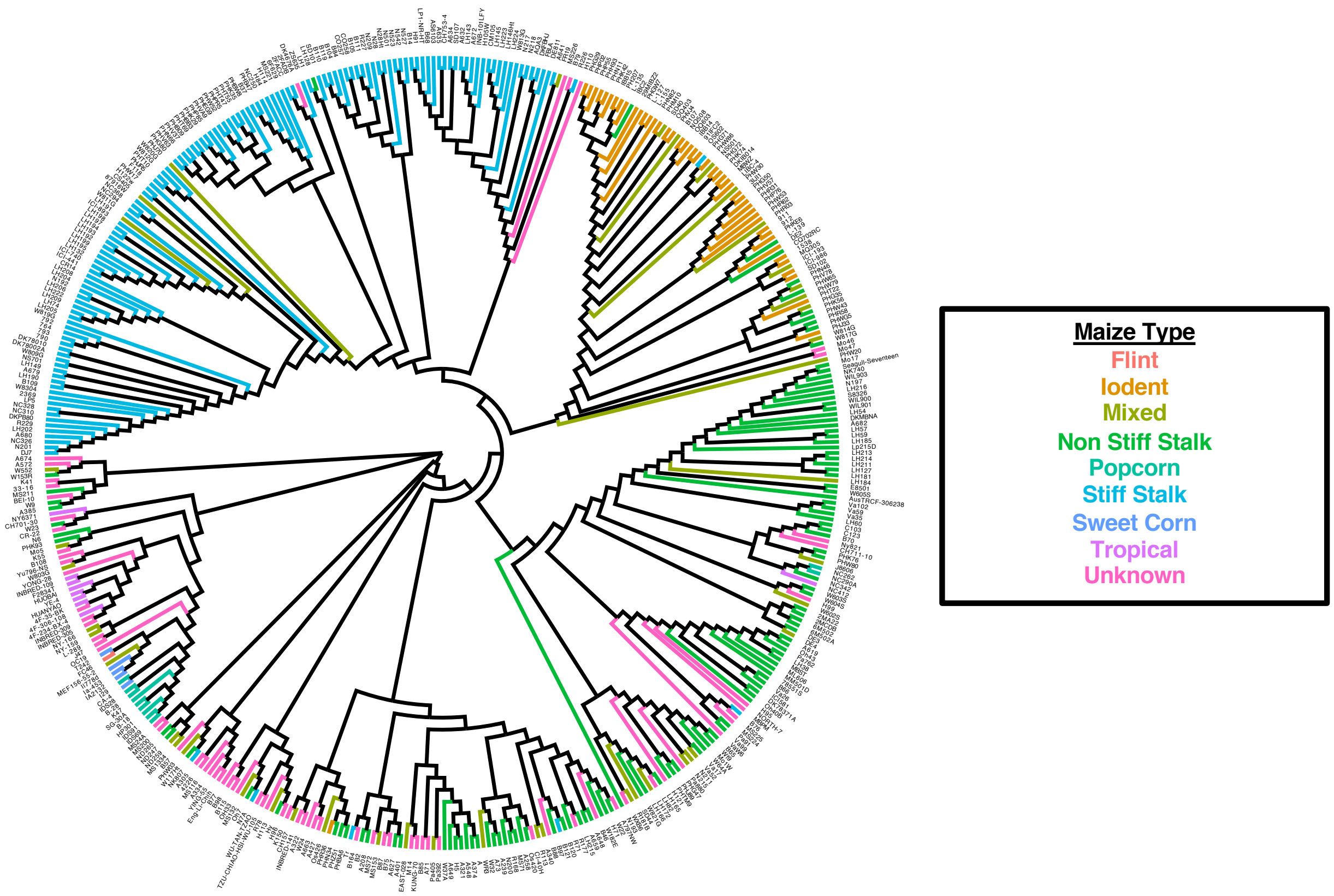

Supplemental Figure 2. Dendrogram of the 446 inbred lines of the WiDiv association panel using 2,412,791 SNPs from WGS data. Distances were based on neighbor-joining clustering and inbreds are colored by maize type identification.

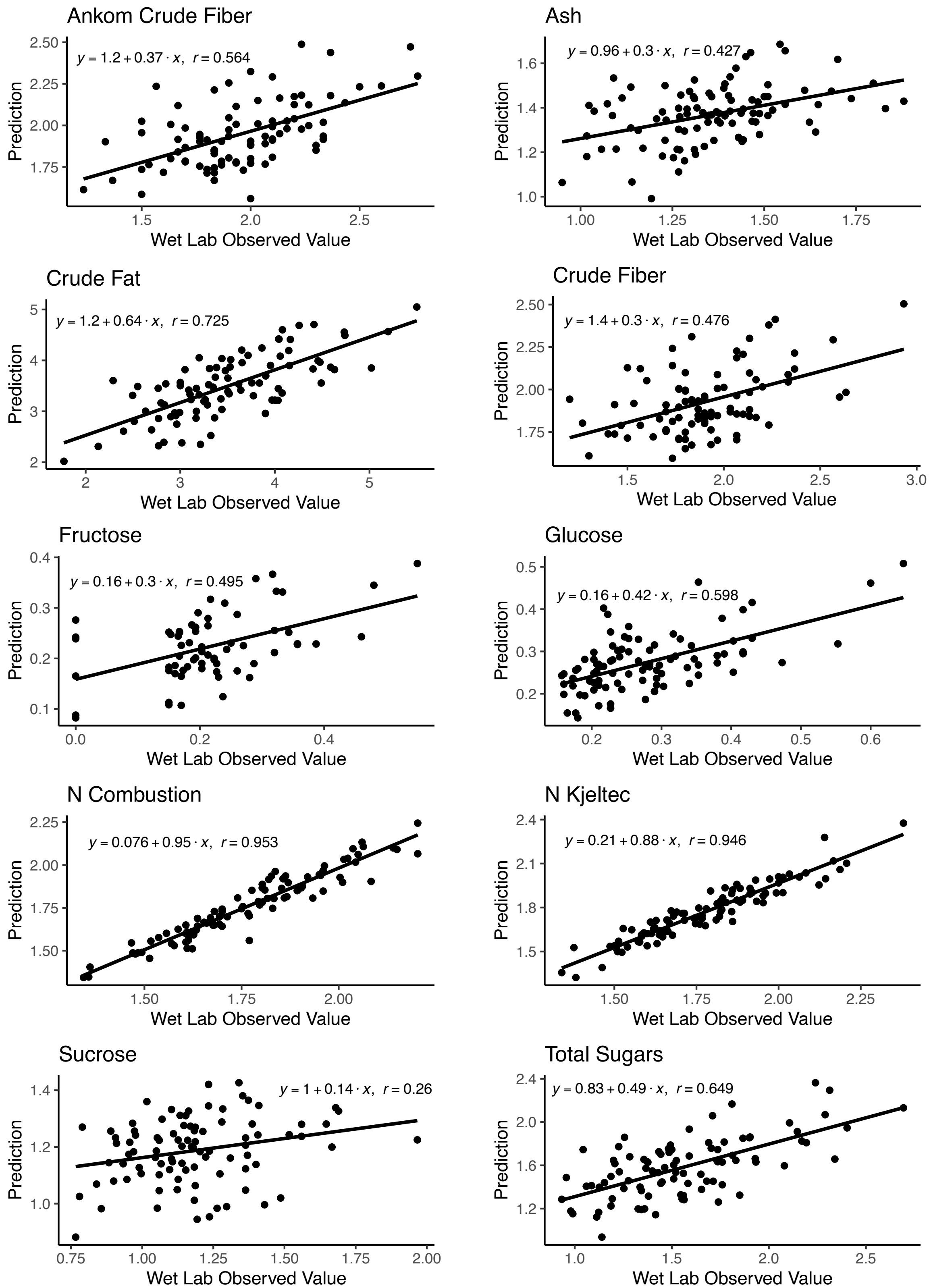

Supplemental Figure 3. Comparison of observed and predicted values for local near-infrared (NIR) spectroscopy prediction equations derived from a subset of 100 inbred lines with diverse spectra profiles.

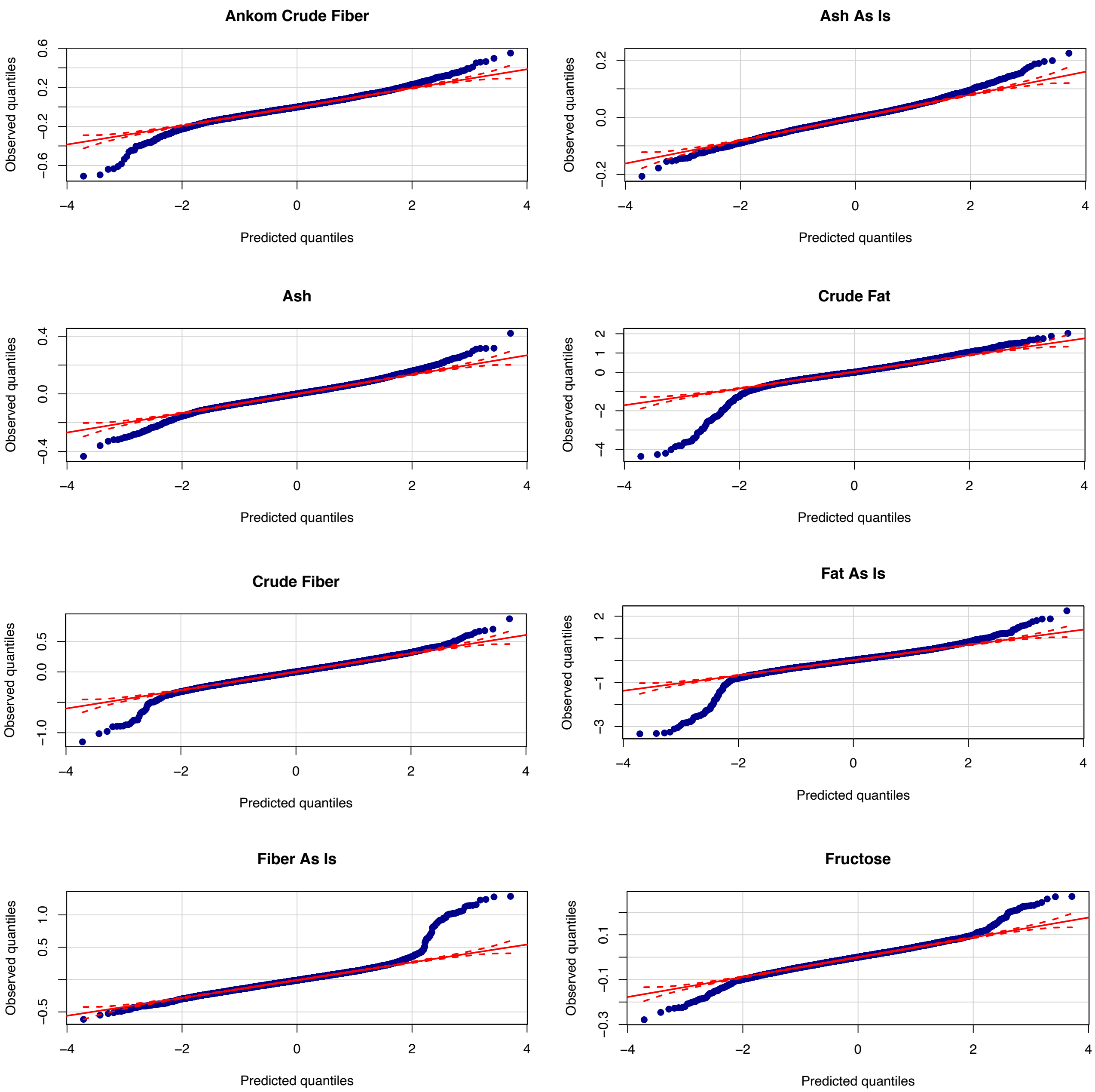

Supplemental Figure 4. Quantile-quantile (Q-Q) plots of the sixteen compositional traits for the random effects model. The red line represents the 1:1 y=x line.

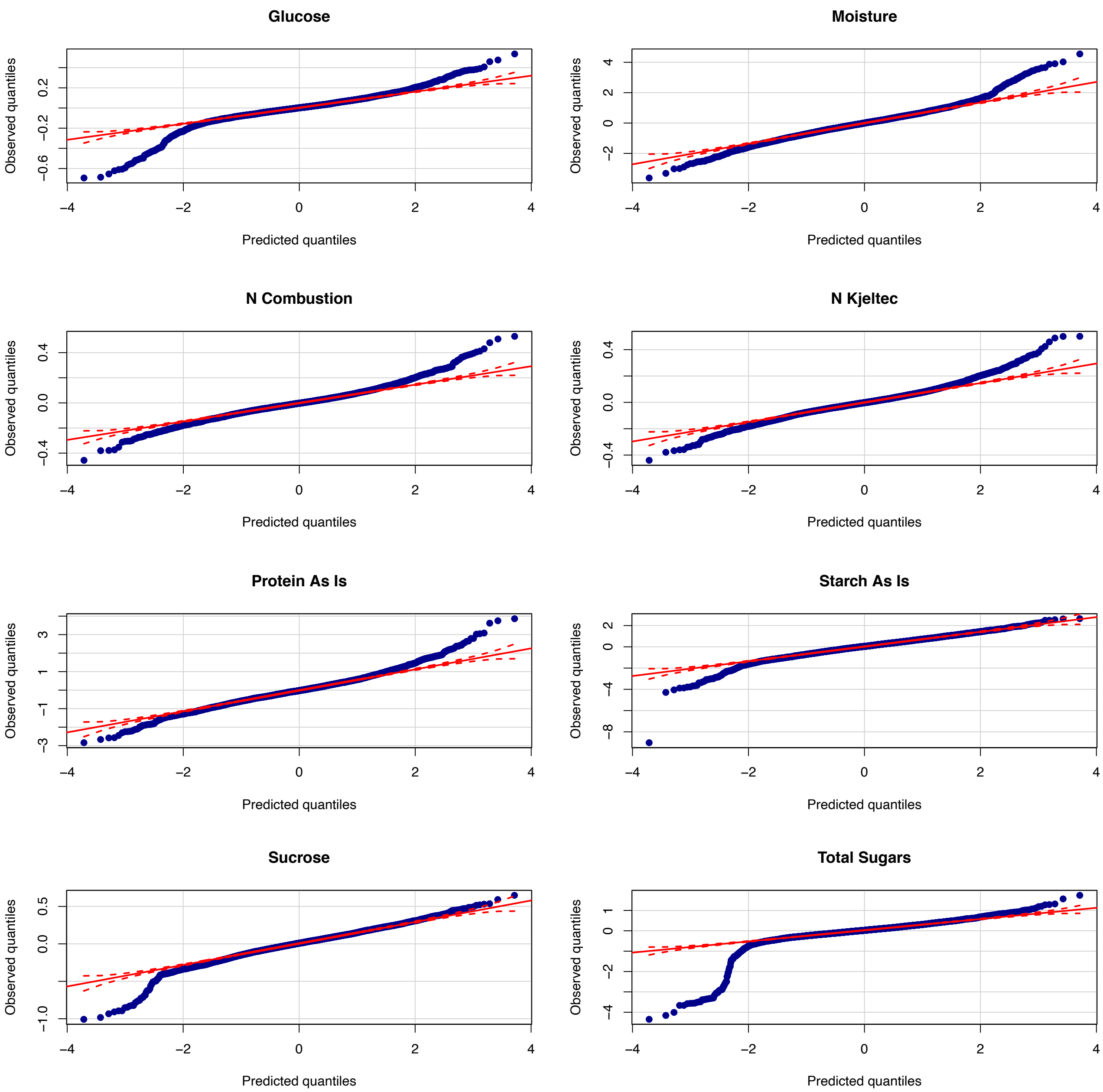

Supplemental Figure 4 continued. Quantile-quantile (Q-Q) plots of the sixteen compositional traits for the random effects model. The red line represents the 1:1 y=x line.

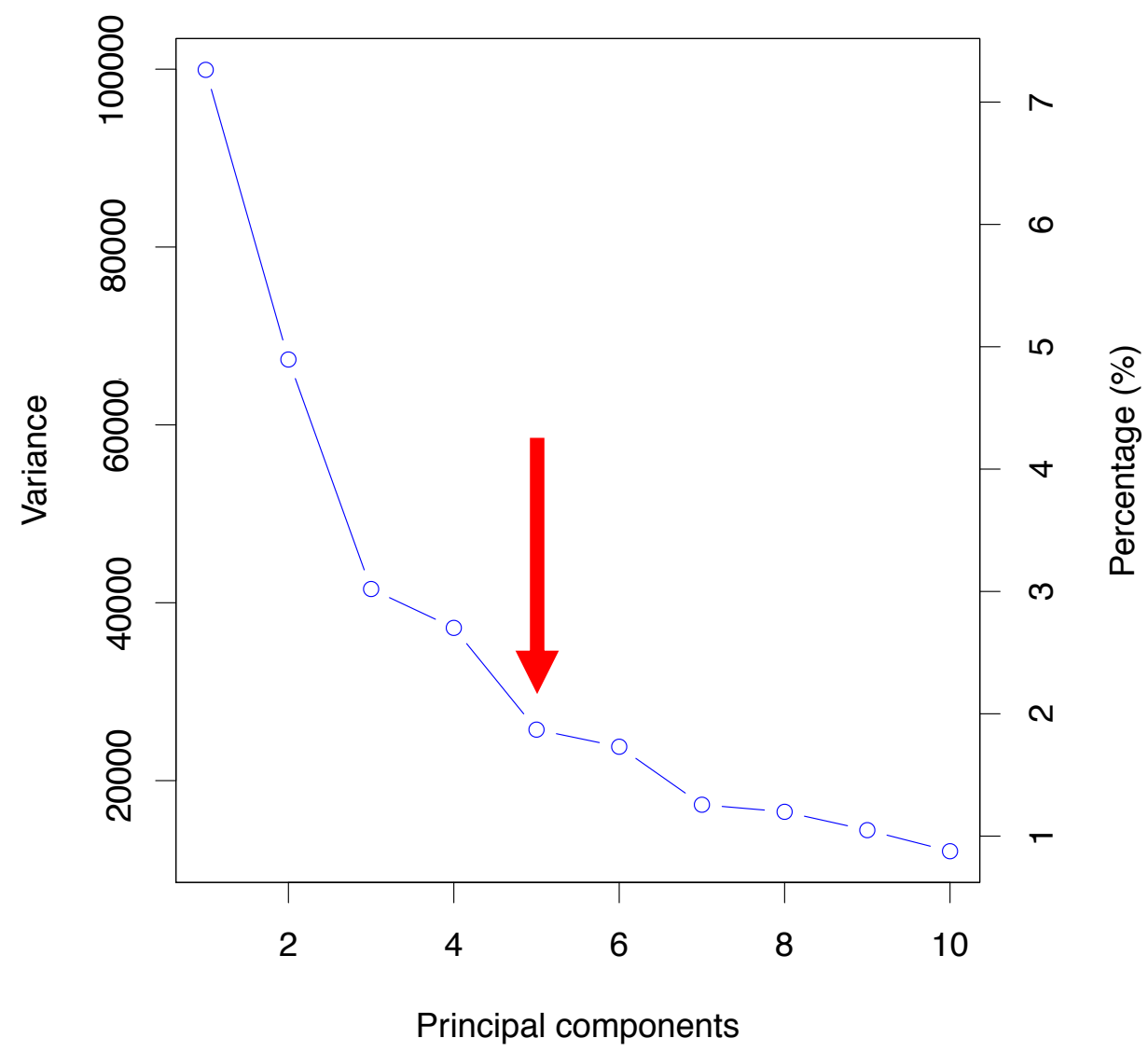

Supplemental Figure 5. Scree plot of eigen values for the principal components from the whole genome sequencing data used on the WiDiv association panel. The red arrow represents the five principal components that were included as a cofactor in the FarmCPU model due to diminished variance explained for an additional principal components beyond n=5.

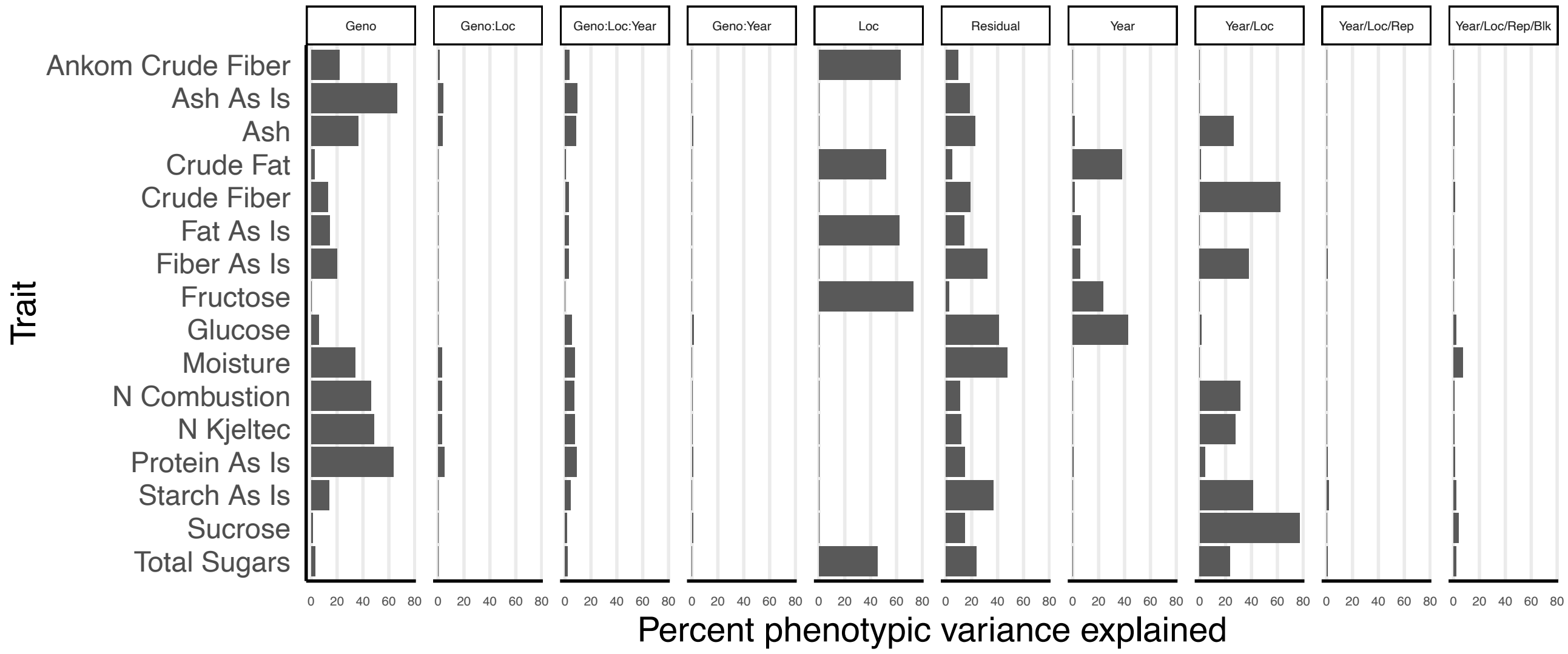

Supplemental Figure 6. Percent of phenotypic variance explained by each factor from the ANOVA of the random effects model for sixteen compositional traits collected from the WiDiv association panel. Geno, genotype, Loc, location, Rep, replication, Blk, block.

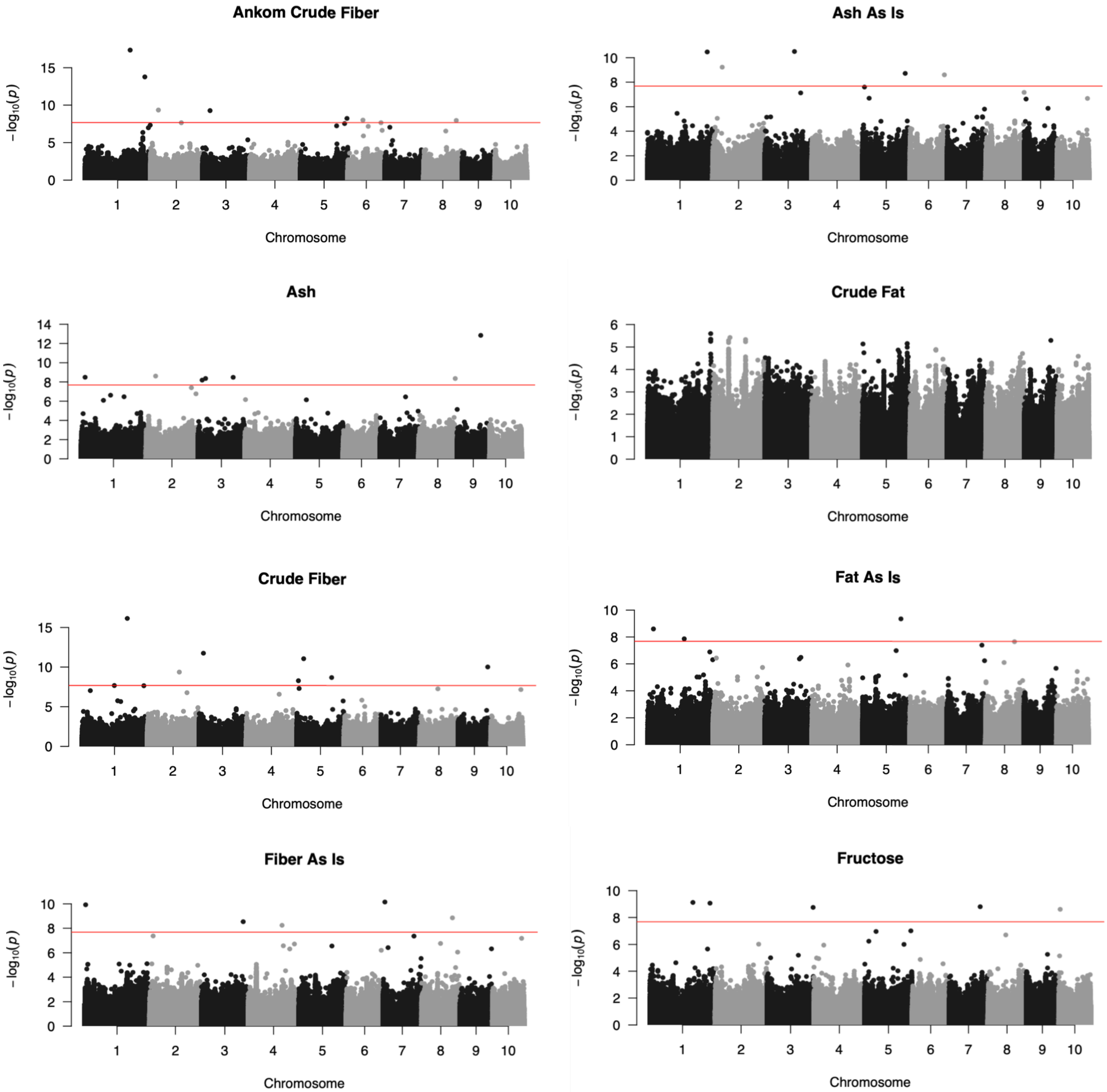

Supplemental Figure 7. Manhattan plots from the GWAS of each compositional trait. Each point represents a single SNP and the solid red line represents the Bonferroni corrected p-value threshold to deem a significant SNP at the 0.05 level (-log(p) = 7.68).

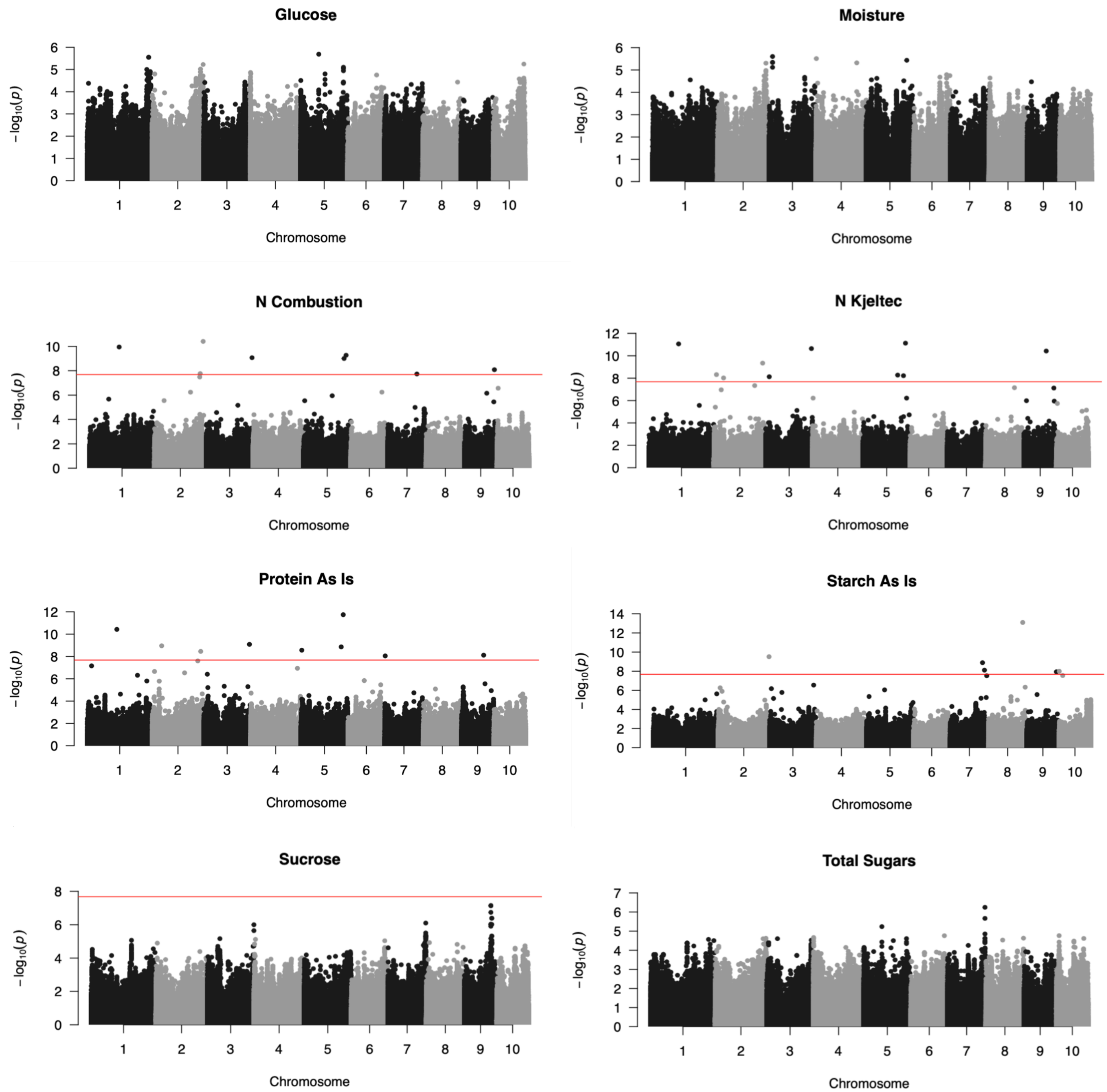

Supplemental Figure 7 continued. Manhattan plots from the GWAS of each compositional trait. Each point represents a single SNP and the solid red line represents the Bonferroni corrected p-value threshold to deem a significant SNP at the 0.05 level (-log(p) = 7.68).
